# Morphological and phylogenetic analyses of *Gonatobotryum* (*Dothideaceae*, *Dothideales*), and the description of a new species from a mangrove sediment in Brazil

**DOI:** 10.1007/s42770-026-02052-1

**Published:** 2026-08-18

**Authors:** Karen B. E. Carmo, Thiago G. Carvalho, Camila S. Oliveira, Esmael A. Vital, Gabriel G. Barreto, Luis F. P. Gusmão, Melissa F. Landell, Pedro H. Félix-Oliveira, Cristina M. Souza-Motta, Layanne O. Ferro, Jadson D. P. Bezerra

**Affiliations:** 1 Laboratório de Micologia (LabMicol), Instituto de Patologia Tropical E Saúde Pública, Universidade Federal de Goiás, Rua 235, S/N, Setor Universitário, Goiânia, GO 74605-050 Brazil; 2 Programa de Pós-Graduação em Biologia da Relação Parasito-Hospedeiro (PPGBRPH), Instituto de Patologia Tropical E Saúde Pública, Universidade Federal de Goiás, Rua 235, S/N, Setor Universitário, Goiânia, GO 74605-050 Brazil; 3 Departamento de Micologia Prof. Chaves Batista, Universidade Federal de Pernambuco, Av. Prof. Moraes Rego, S/N, Centro de Biociências, Cidade Universitária, Recife, PE 50670-901 Brazil; 4 Programa de Pós-Graduação em Biologia de Fungos (PPGBF), Departamento de Micologia Prof. Chaves Batista, Universidade Federal de Pernambuco, Av. Prof. Moraes Rego, S/N, Centro de Biociências, Cidade Universitária, Recife, PE 50670-901 Brazil; 5 Instituto de Ciências Biológicas e da Saúde, Universidade Federal de Alagoas, Campus A.C. Simões, Maceió, AL 57072-900 Brazil; 6 Departamento de Biociências, Centro de Ciências Agrárias - Campus II, Universidade Federal da Paraíba, Rodovia PB 079 - Km 12, Areia, Paraíba 58397-000 Brazil; 7 Departamento de Ciências Biológicas, Universidade Estadual de Feira de Santana, Av. Transnordestina S/N, Novo Horizonte, Feira de Santana, Bahia 44036-900 Brazil

**Keywords:** Biodiversity, Brazilian fungi, Systematics, Taxonomy, New taxa

## Abstract

*Gonatobotryum* is an anamorphic fungus reported mainly as a saprophyte, parasite, or endophyte. The genus has traditionally been identified based on morphological features, with its phylogenetic placement remaining uncertain due to the lack of molecular data. During the analysis of fungi obtained from mangrove sediments in Brazil, nine isolates were morphologically identified as *Gonatobotryum* and subsequently analysed using the internal transcribed spacer (ITS) region, including the intervening 5.8S nRNA gene and the nuclear ribosomal RNA large subunit (LSU) gene, leading to the description of a new species, *Gonatobotryum mangrovei*. Additionally, the type material of *Gonatobotryum bahiense* was re-evaluated regarding its phylogenetic placement. Here, we propose a new combination, *Ophiostoma bahiense*, to place this species within the genus. Our results clarify the phylogenetic position of *Gonatobotryum* and expand the current understanding of fungi in mangrove environments.

**Supplementary Information:**

The online version contains supplementary material available at 10.1007/s42770-026-02052-1.

## Introduction

The genus *Gonatobotryum* is a hyphomycete fungus introduced by Saccardo [1], with *G. fuscum* (Sacc.) Sacc. as the type species. It was found on decaying *Quercus* wood in Italy and was previously treated as *Gonatobotrys fuscus* Sacc., a genus morphologically similar to *Gonatobotryum* [2]. *Gonatobotrys* Corda is currently placed in the family *Ceratostomataceae* (*Coronophorales*, *Ascomycota*) [3]. The morphology of *Gonatobotryum* is characterised by erect and unbranched conidiophores, nodulose, denticulate conidiogenous cells extending several times, and conidia ovoid, which are key features to separate its species from genera morphologically similar, *e.g. Nematogonum* (*incertae sedis*) and *Gonatobotrys* [4–6], and *Neonematogonum* (*Helotiales*) [7]. In the Index Fungorum and MycoBank databases, there are 11 records of species names under *Gonatobotryum* (accessed March 27, 2026), with *Gonatobotryum piceae* Dörfelt & A.R. Schmidt being the most recently described species, occurring on the hypocotyls and cotyledons of fossil *Picea* in amber from Russia [8]. All *Gonatobotryum* species were initially described solely based on the morphology of the type material, with no DNA sequences available, making it challenging to verify their classification using modern taxonomy. As a result, the genus *Gonatobotryum* has been classified as *incertae sedis* within *Ascomycota* [3].

Recently, based on ITS and LSU rDNA sequences from new isolates, the genus was tentatively assigned to *Dothioraceae* (*Dothideales*) [9]. Nonetheless, *Dothioraceae* was synonymised with *Dothideaceae* based on morphological and phylogenetic inferences within *Dothideales*, an order that currently comprises four families (*Dothideaceae*, *Neocelosporiaceae*, *Saccotheciaceae*, and *Zalariaceae*) [10, 11]. The taxonomic tradition of the genus has remained centred on morphological characters, particularly conidiogenesis [4, 5, 9, 12]. One of the most important studies of this genus was published by Walker & Minter [5], which used light and scanning electron microscopy to assess the morphology of type materials. Walker & Minter [5] studied conidiogenesis in *Gonatobotryum* in detail and, among the eight species treated, accepted three as members of the genus (*G. apiculatum*, *G. fuscum*, and *G. parasiticum*). The doubtful or excluded species were treated in other genera or as *incertae sedis*. *Gonatobotryum* species are commonly reported to be mycoparasitic, although they can also act as saprobes and endophytes. As saprobes, they have been recorded from soil and decaying wood [5]. *Gonatobotryum parasiticum* is parasitic on other fungi and is considered a probable contact mycoparasite [5]. Other reports include *G. fuscum* as a mycoparasite of several *Ceratocystis* species [13]; *G. bimorphosporum* on young leaves of *Carissa carandas* in India [14]; *G. apiculatum* causing leaf spot and blight on *Sinowilsonia henryi* and *Hamamelis* spp. in China [9]; and *G. piceae* from fossil seedlings of *Picea* in Russia [8].

During a survey of mangrove sediment in Brazil, nine isolates were obtained and morphologically identified as *Gonatobotryum*. Subsequently, these isolates were studied using multilocus phylogenetic analyses. Additionally, we examined the type material of *Gonatobotryum bahiense* Bat. [15], deposited in the URM culture collection in Brazil, and performed morphological and phylogenetic analyses to assess its current phylogenetic placement.

## Materials and methods

### Fungal isolates

Fresh *Gonatobotryum* isolates were obtained from the mangrove sediment collected in the Área de Proteção Ambiental Santa Rita (APA Santa Rita) in the municipality of Marechal Deodoro, state of Alagoas, Brazil (09°42′51,72"S 35°49′10,77"W), during the dry season in February 2025. For the isolation of fungi, we followed the methods described by Clark [16], modified by using 1 g of sediment suspended in water containing chloramphenicol (0.5 g/L). This suspension was considered as the 10⁻^1^ dilution, from which the subsequent dilution (10⁻^2^) was prepared. A 1 mL aliquot was transferred to Petri dishes containing the culture media Soluble Starch-Sea Water Agar (SSA: 10 g soluble starch, 1 g yeast extract, 18 g agar, 38 g sea salt, and 1 L of distilled water) and Sea Water Agar (SW1: 5 g glucose, 1 g yeast extract, 18 g agar, 38 g sea salt, and 1 L of distilled water) (based on González et al. [17]; Nakagiri & Tubaki [18]), both supplemented with chloramphenicol (0.5 g/L) to inhibit bacterial growth. The plates were incubated in the dark at 25 ± 2 °C for up to 7 days to count colony-forming units (CFU), and representative fungi were selected for isolation. The isolates were cultured on potato dextrose agar (PDA) and incubated in the dark at 25 °C for 7 to 10 days for further analysis. The isolates obtained in our study are deposited in the working collection of Jadson Bezerra (JB) and in the Coleção de Culturas de Fungos da Universidade Federal de Goiás (FCCUFG) housed at the Laboratório de Micologia (LabMicol) of the Instituto de Patologia Tropical e Saúde Pública (IPTSP) of the Universidade Federal de Goiás (UFG, Goiânia, Brazil). Holotypes, as metabolically inactive cultures, are deposited in the University Recife Mycology (URM) culture collection (Micoteca URM, Universidade Federal de Pernambuco, Recife, Brazil), and permanent ex-holotype microscopic slides are deposited in the UFG herbarium (Herbário UFG, Goiânia, Brazil). Ex-type strains are deposited in the FCCUFG and URM culture collections. Additionally, we analysed the type strain URM 309, identified as *G. bahiense*, and deposited in the URM culture collection (Recife, Brazil) [15].

### DNA extraction, PCR, and sequencing

For genomic DNA extraction, the Wizard® Genomic DNA Purification Kit (Promega, USA) was used according to the manufacturer’s instructions. PCR amplifications for internal transcribed spacer regions (ITS), part of the nuclear ribosomal large subunit (LSU), translation elongation factor 1-alpha (*tef1*), β-tubulin (*tub2*), and RNA polymerase II, the second largest subunit (*RPB2*), using the primer sets ITS4/ITS5, LR5/LR0R, EF-983F/EF-2218R, Bt2a/Bt2b, and RPB2-5F/RPB2-7cR, respectively. References for primers, PCR conditions, sequencing, and sequence editing were performed as described by Ferro et al. [19]. The sequences obtained in this study have been deposited in the NCBI GenBank database (Table 1).

**Table 1:** GenBank accession numbers of sequences used in this study. The alignments for *Gonatobotryum* were obtained from Nascimento et al. [21], and here are listed only the new sequences included in our study. Sequences in bold were generated in this study. Ex-type strains are marked with (T)

Species	Strain	GenBank numbers				
		ITS	LSU	*tub2*	*RPB2*	*tef1*
*Gonatobotryum apiculatum*	CBS 182.68 = ATCC 18363 = IMI 131792 = MUCL 11459 = FMR 20101 = UW 200	MH859103	MH870816	-	-	-
***Gonatobotryum mangrovei* sp. nov**	**JB5411**	**PZ162102**	**-**	**-**	**PZ231493**	**-**
	**JB5412**	**PZ162103**	**-**	**-**	**PZ231494**	**-**
	**JB5413**	**PZ162104**	**-**	**-**	**PZ231495**	**-**
	**JB5414**	**PZ162105**	**-**	**-**	**PZ231496**	**-**
	**JB5415**	**PZ162106**	**-**	**-**	**-**	**-**
	**FCCUFG 224 = JB5416**	**PZ162098**	**PZ162107**	**PZ167275**	**PZ231497**	**-**
	**URM 9435**^ T^** = FCCUFG 223 = JB5417**	**PZ162099**	**PZ162108**	**PZ167274**	**PZ231498**	**PZ231491**
	**JB5418**	**PZ162100**	**PZ162109**	**PZ167276**	**-**	**PZ231492**
	**JB5419**	**PZ162100**	**PZ162110**	**-**	**-**	**-**
*Gonatobotryum* sp.	YZU 181227	MK895978	MK895979	-	-	-
*Gonatobotryum* sp.	SX1	KJ620838	-	-	-	-
*Gonatobotryum* sp.	CB21-775	OR803714	-	-	-	-
*Gonatobotryum* sp.	CB21-774	OR803713	-	-	-	-
*Gonatobotryum* sp.	CB21-560	OR803513	-	-	-	-
*Gonatobotryum* sp.	CB21-548	OR803502	-	-	-	-
*Ophiostoma allantosporum*	CMW 163	OM501464	OM514792		-	-
***Ophiostoma bahiense***	**URM 339**^ T^** = CBS 141532**	**MH384821**	**MH370604**	**MH370608**	**-**	**-**
*Ophiostoma bicolor*	CFCC 57909	PQ166533	-	PQ166480	-	-
*Ophiostoma clavatum*	CMW 37983	OM501477	OM514805	KU094705	-	-
	CBS 135.51^ T^	-	MH868301	-	-	-
*Ophiostoma conicolum*	CBS 127.89	OM501478	AF155689		-	-
*Ophiostoma ips*	CFCC 71137	PQ623411	-	PQ619702	-	-
*Ophiostoma longiconidiatum*	CMW 17574^ T^	NR_136983	EF408558	-	-	-
*Ophiostoma macroclavatum*	4SSW	KY568130	-	-	-	-
*Ophiostoma minus*	CCMA 12	AY934511	-	-	-	-
*Ophiostoma multiannulatum*	CBS 139.36	MH855740	MH867252	-	-	-
*Ophiostoma novae-zelandiae*	UAMH 9559^ T^	NR_147576	-	KT381293	-	-
	CIEFAP 423	-	KT362226	KT381292	-	-
*Ophiostoma palustre*	CMW 44403^ T^	NR_147592	-	KX273409	-	-
	WN 2018	MW028168	MW028136	-	-	-
*Ophiostoma piceae*	CMW 25034	KU184441	-	KU184312	-	-
*Ophiostoma pityokteinis*	CBS 144877	MH837044	-	-	-	-
*Ophiostoma pluriannulatum*	CMW 22803	DQ539508	-	-	-	-
	CMW 75	-	DQ294365	DQ296085	-	-
*Ophiostoma subannulatum*	CBS 188.86	KU756601	AF135574	-	-	-
*Ophiostoma ulmi*	CBS 106.63	MH858227	MH869829	-	-	-
*Ophiostoma vilosum*	N2016 1572/2/1	MH055647	-	-	-	-
*Sporothrix mexicana*	CBS 120341	NR_147564	KX590887	AM498344	-	-

### Phylogenetic analyses

Phylogenetic analyses were performed using our sequences and reference sequences for genera included in *Dothideales*, as defined in recent studies [3, 20]. The type strain URM 339 was re-evaluated using sequences from studies of *Ophiostoma* [21, 22]. The sequences were aligned using the MAFFT v. 7 online interface [23] and manually edited using MEGA v. 7 software [24]. We performed a maximum likelihood (ML) analysis for each gene and the combined matrix to determine the phylogenetic placement of our isolates. The ML analysis was performed using IQ-TREE v. 1.6.12 [25] and RAxML HPC-BlackBox v. 8.2.12 [26]. The RAxML-HPC BlackBox ML analysis was performed using the default system options on the CIPRES Science Gateway [27] (http://www.phylo.org/) with 1,000 bootstrap replicates. The IQ-TREE ML analysis involved 5,000 replicates, and the ultrafast bootstrap (UFboot2) method was used to estimate branch support [28]. The ModelFinder software, included in IQ-TREE v. 1.6.12 [29], was used to determine the partitioning strategy and models based on the Akaike information criterion. The combined datasets were also analysed using Bayesian inference (BI) with MrBayes v. 3.2.7a [30] on the CIPRES Science Gateway, employing the same models and partitions as in the ML analysis (IQ-TREE). The BI analysis was conducted with 50 M generations and a 25% burn-in, with chains sampled every 1,000 generations. The resulting phylogenetic trees were visualised using FigTree v. 1.4.4 [31]. Values ≥ 0.95 BI posterior probability (BPP) and bootstrap support (BS) from IQ-TREE UFboot2-BS and RAxML-BS analyses (both BS ≥ 70%) were plotted near the nodes. The final combined alignments were deposited in Figshare (10.6084/m9.figshare.31919979).

### Morphology

For macroscopic characterisation of the colonies, our isolates were inoculated onto malt extract agar (MEA), oat agar (OA), and potato dextrose agar (PDA) [32], contained in 90 mm Petri dishes and incubated at 25 ± 2 °C in a 12 h dark/light cycle for 7 days (*Gonatobotryum*) or 14 days (*Ophiostoma*). Colony characteristics, including diameter, margin appearance, texture, presence of aerial mycelium, and colony and reverse colour, were evaluated. Rayner’s colour scale [33] was used to determine colony colour. The morphological features were visualised after 7 and 14 days on MEA, PDA, and OA culture media. Microscopic characterisation was performed using Riddell’s microculture method [34] on PDA, allowing detailed observation of structures such as conidia, conidiophores, and conidiogenous cells, and their shape, size, and colour. The images were captured with a Leica DM2500 LED microscope having differential interference contrast (DIC) optics and equipped with a camera DFC7000T and processed using Leica Application Suite X (LAS X, v. 3.9.1.28433) at the Centro Multiusuário de Pesquisa de Bioinsumos e Tecnologias em Saúde (CMBiotecs/IPTSP/UFG, Goiás, Brazil).

## Results

**Fig. 1:**
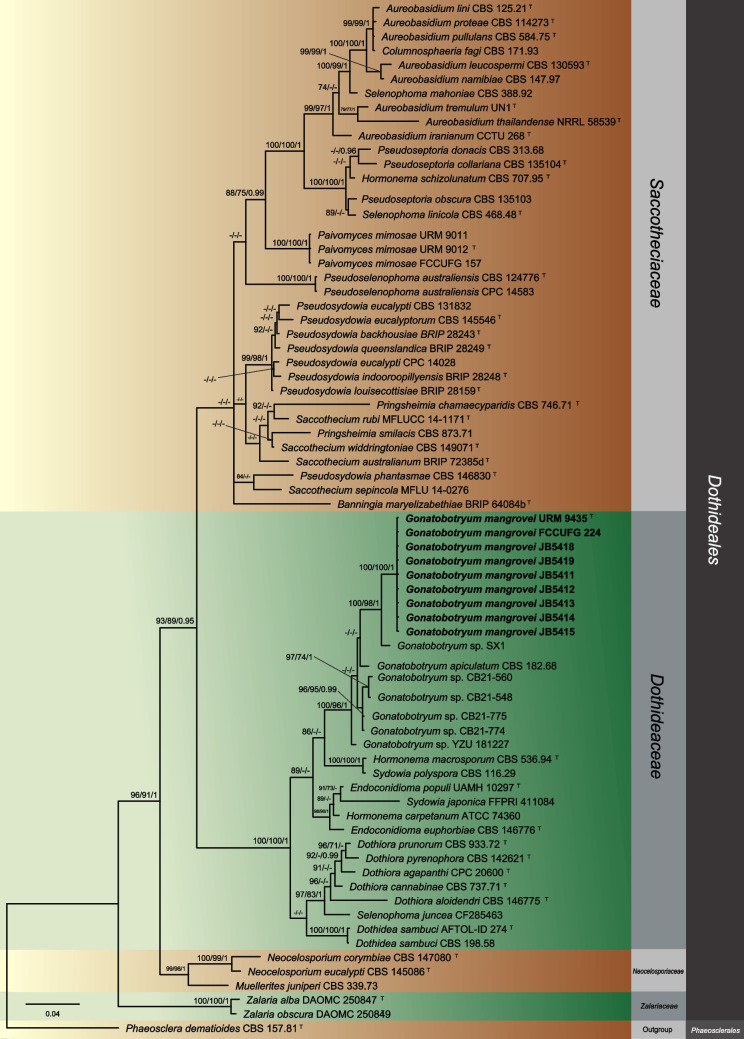
Bayesian inference tree based on combined ITS and LSU sequences of the new species *Gonatobotryum mangrovei* sp. nov. and related taxa in *Dothideaceae* (*Dothideales*). The new species is shown in bold. Support values for ML-BS and IQ-TREE-BS ≥ 70% (UFboot2/RAxML) and BPP ≥ 0.95 are indicated near the nodes, and (–) indicates support below the threshold values. Type strains are marked with (T). The tree was rooted to *Phaeosclera dematioides* (CBS 157.81) (*Phaeosclerales*, *Dothideomycetes*)

We analysed nine *Gonatobotryum* isolates (Table 1). Based on morphological features and phylogenetic inferences from ITS and LSU rDNA sequences, they are described below as a new species. The combined matrix (ITS and LSU) comprised sequences from 72 strains, including the outgroup *Phaeosclera dematioides* CBS 157.81 (*Phaeoscleraceae*, *Phaeosclerales*) (Fig. 1), and 1,284 characters, including gaps (567 characters for ITS and 717 for LSU), of which 845 were conserved, 429 variable, and 311 parsimony-informative. The models used for IQTREE ML and BI were GTR + I + G for ITS and SYM + I + G for LSU. For the ML analysis (RAxML-HPC BlackBox), the GTR + I + G model was used, and the matrix contained 516 distinct alignment patterns, with 21.5% undetermined characters or gaps. The final ML optimisation likelihood value of the best tree was −8962.111404, and the estimated base frequencies were: A = 0.249915, C = 0.229542, G = 0.277484, T = 0.243059; substitution rates: AC = 1.436720, AG = 1.800865, AT = 1.954029, CG = 0.571607, CT = 5.258843, GT = 1.000000; gamma distribution shape parameter: α = 0.443580. We conducted combined and individual phylogenetic analyses for each gene (ITS and LSU), and our sequences were placed in a unique, well-supported lineage (ML-BS = 100%, IQ-TREE-BS = 100%, and BPP = 1) related to other *Gonatobotryum* sequences in *Dothideacea* (Fig. 1; Online resources 1 and 2). As the majority of species in our analysis have only ITS and LSU sequences available, we generated *tef1*, *tub2*, and *RPB2* sequences for further phylogenetic analysis of the group.

**Fig. 2:**
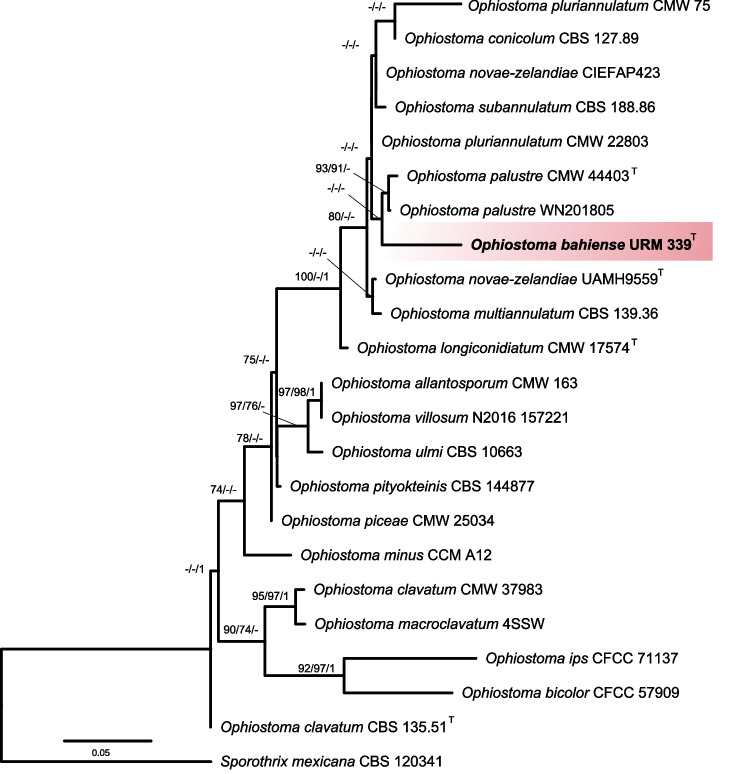
Maximum Likelihood (ML) phylogenetic tree based on combined ITS, LSU, and *tub2* sequences of the new combination *Ophiostoma bahiense*. The specimens obtained in this study are in bold. Ex-type strains are marked with (T). Confidence values for ML-BS ≥ 70% (UFboot2/RAxML) and BPP ≥ 0.95 are included near the nodes, and the “-” indicates statistical support below the threshold values. The tree was rooted to *Sporothrix mexicana* (CBS 120341)

**Fig. 3:**
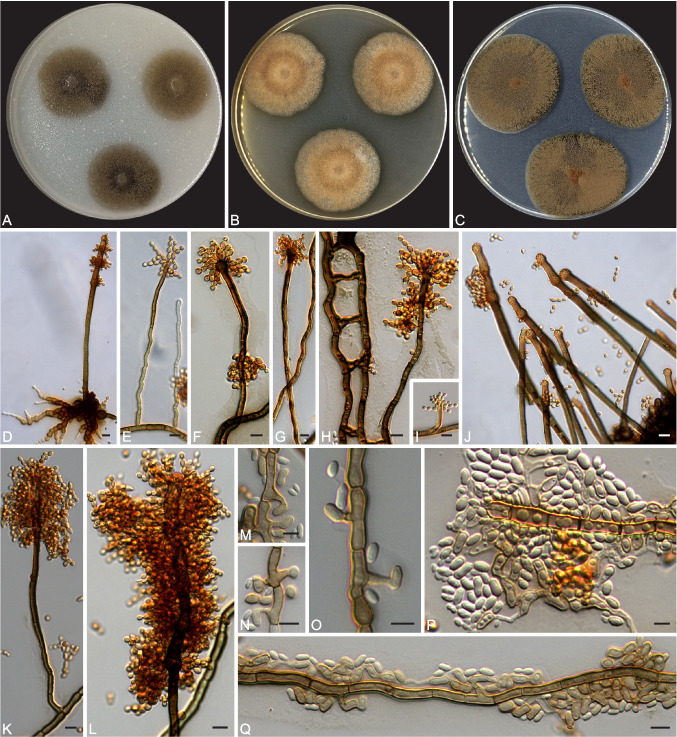
*Gonatobotryum mangroveii* sp. nov. (ex-holotype URM 9435). **A**. Colony on OA. **B**. Colony on MEA. **C**. Colony on PDA. **D**-**G**, **I**-**K**. Conidiophores, ramoconidia, and conidia. **H**. Anastomoses, conidiophores, ramoconidia, and conidia. **L**. Detail of conidiogenous cells producing ramoconidia and conidia. **M**-**Q**. Aureobasidium-like structures. **P**. Showing some inconspicuous denticles. Scale bars: 10 µm

The strain URM 339 was initially circumscribed as *Gonatobotryum bahiense*; however, our BLASTn searches indicated that it is related to *Ophiostoma* species. Phylogenetic analyses based on ITS sequences from all *Ophiostoma* sequences (data not shown) placed strain URM 339 within the *O. pluriannulatum* species complex. Based on morphological features and phylogenetic inferences from ITS, LSU, and *tub2* sequences, we propose the new combination *Ophiostoma bahiense* to accommodate this species. The combined matrix (ITS, LSU, and *tub2*) comprised sequences from 23 strains belonging to the *O. pluriannulatum* species complex and representatives of the other *Ophiostoma* complexes, including the outgroup *Sporothrix mexicana* (CBS 120341) (Fig. 2), and 1,731 characters, including gaps (622 characters for ITS, 736 for LSU, and 373 for *tub2*), of which 1,364 were conserved, 300 variable, and 181 parsimony-informative. The models used for IQTREE ML and BI were GTR + I + G for both genes. For the ML analysis (RAxML-HPC BlackBox), the GTR + I + G model was used, and the matrix contained 380 distinct alignment patterns, with 45.11% undetermined characters or gaps. The final ML optimisation likelihood value of the best tree was −4911.628965 and the estimated base frequency were: A = 0.207897, C = 0.295963, G = 0.289751, T = 0.206389; substitution rates: AC = 1.905602, AG = 3.659128, AT = 2.710493, CG = 1.637065, CT = 12.440419, GT = 1.000000; gamma distribution shape parameter: α = 0.395955. We conducted combined (Fig. 3) and individual phylogenetic analyses for each gene (ITS, LSU, and *tub2*; data not shown), and our sequences formed a unique lineage related to *Ophiostoma* sequences.

### Taxonomy

***Gonatobotryum*** Sacc., Michelia 2(no. 6): 24 (1880).

*Type species: Gonatobotryum fuscum* (Sacc.) Sacc.

*Accepted species: Gonatobotryum apiculatum* (Peck) S. Hughes, *G. bimorphosporum* M. Jacob & Bhat, *G. parasiticum* (Thaxt.) Jane Walker & Minter, *G. piceae* Dörfelt & A.R. Schmidt, and the new species described below.

***Gonatobotryum mangrovei*** K.B.E. Carmo, T.G. Carvalho, L.O. Ferro & J.D.P. Bezerra, **sp.* nov.*** Fig. 3

*MycoBank:* MB 863147.

*Etymology:* Named in reference to the substrate, a mangrove sediment, from which the fungus was obtained in Brazil.

*Type:* **Brazil**, Alagoas state, Marechal Deodoro municipality, Área de Proteção Ambiental (APA) de Santa Rita, Lagoa Manguaba (09°42′51.72"S 35°49′10.77"W), isolated from mangrove sediment, February 2025, collected by M.F. Landell, E.A. Vital, and V. Petra, isolated by K.B.E. Carmo & T.G. Carvalho [**holotype** URM 9435HT (metabolic inactive state), microscopic slide ex-holotype UFG 58681, culture ex-holotype URM 9435 = FCCUFG 223].

*Description:* On PDA. *Mycelium* immersed and superficial, composed of septate hyphae, constricted at the septa, inflated, root-like aspect, branched, sometimes anastomoses, pale brown to dark brown, subhyaline at the tips, 4 − 6 (− 8) μm wide. *Conidiophores* macronematous, mononematous, flexuous to erect, unbranched, septate, brown, becoming pale brown or subhyaline towards the apex, nodose, occasionally producing a foot-like cell and rhizoid-like structures at base, (28 −) 115 − 215 (− 232) × (3 −) 4 − 7 (− 9) µm. *Conidiogenous cells* polyblastic, denticulate, indeterminate, integrated, terminal or intercalary, light to dark brown, spherical to subspherical, (4.5 −) 8 − 10 (− 11.5) × (4.5 −) 8 − 8.5 (− 10) µm. *Conidial secession* schizolytic. *Ramoconidia* ellipsoidal to cylindrical, aseptate, brown, smooth-walled, 5.5 − 6 × 3 − 4 µm. *Conidia* acropleurogenous, blastocatenate, globose to subglobose, ellipsoidal to cylindrical, tapering to a truncate hilum at the ends dark brown, smooth-walled, hyaline when young to dark brown with age, arranged in fasciculate clusters from conidiogenous loci; *primary conidia* arising from ramoconidia, 4.5 − 5.5 × 3 − 4 µm; *secondary conidia* arising from primary conidia, (3 −) 4 − 4.5 (− 5.5) × (3 −) 3.5 − 4 (− 5) µm. *Synanamorph* aureobasidium-like. *Conidiophores* micronematous, reduced to conidiogenous cells monoblastic and polyblastic-synchronous, inconspicuous denticulate, intercalary, and terminal discrete or integrated, brown, (13 −) 15 − 20 (− 26.5) × (5.5 −) 7 − 9 (− 10) μm. *Conidia* solitary, ellipsoidal to oblong, aseptate, hyaline to brown, smooth, sometimes guttulate, (4.5 −) 10 − 12.5 (− 13.5) × (2 −) 4 − 5 (− 5.5) μm; accumulating in white layers or masses.

*Culture characteristics:* On MEA, colonies are circular with a regular edge, texture floccose with aerial mycelia forming a velvety halo, colour buff with a cinnamon halo, and reverse buff with a dark brick and ochreous edge, reaching 35.7 mm diam. On OA, colonies are circular with a regular edge, texture fasciculate to slightly coriaceous, sporulation moderate, colour hazel, and reverse vinaceous buff to sepia, growing up to 30 mm. On PDA, colonies are circular with a regular edge, texture fasciculate, sporulation intense, colour fawn, and reverse pale purplish grey with a violaceous-black halo and an isabelline edge, growing up to 43.8 mm.

*Other materials analysed:* **Brazil**, Alagoas state, Marechal Deodoro municipality, Área de Proteção Ambiental (APA) de Santa Rita, Lagoa Manguaba (09°42′51.72"S 35°49′10.77"W), isolated from mangrove sediment, February 2025, collected by M.F. Landell, E.A. Vital, and V. Petra, isolated by K.B.E. Carmo & T.G. Carvalho (isolates FCCUFG 224 = JB5416, JB5411, JB5412, JB5413, JB5414, JB5415, JB5418, and JB5419).

*Notes:* Based on current phylogenetic analyses (Fig. 1; Online resources 1 and 2), the available sequences of *Gonatobotryum* spp. are placed as a distinct lineage among genera in *Dothideaceae* (*Dothideales*). BLASTn searches of the ITS and LSU sequences generated in this study revealed a close relationship (95–96%) with *G. apiculatum* isolates, the only species for which sequence data are available in GenBank. We currently do not have DNA sequences available for the type species of this genus, *G. fuscum*, or any other type material. All *Gonatobotryum* species were historically established solely based on morphological characteristics. *Gonatobotryum mangrovei* differs from the accepted species of *Gonatobotryum* by the size of conidiophores, conidiogenous cells, and conidia, the latter here described as ramoconidia, primary, and secondary conidia (Table 2). Phylogenetically, we observed that the isolate YZU 181227, previously identified as “*G. apiculatum*” by Gao et al. [9], occupied a distinct position in our phylogenetic analyses, suggesting that it does not represent *G. apiculatum*. To ensure a more stable application of the name *G. apiculatum*, we treated the strain CBS 182.68 as a reference material for *G. apiculatum*, since it is from the same geographic region (North America) as the material analysed by Hughes [35] and Walker & Minter [5]. The remaining *Gonatobotryum* isolates with available DNA sequences are here treated as *Gonatobotryum* sp. until new analyses resolve these lineages.

**Table 2:** Main morphological features and habitat of accepted *Gonatobotryum* species

Species	Conidiophores	Conidiogenous cells	Conidia	Observations	References
*G. apiculatum*	Light to dark brown, roughened towards the base, smooth; 250–1500 × 5–12 µm	Light to dark brown, smooth, with a swollen part; 10–24 × 7–19 µm	Light brown, branched chains, distal conidia smaller than the proximal ones, smooth, elliptical to globose, with conspicuous denticles, rhexolytic; 3–13 × 2–7 µm	*Anacardium*, *Hamamelis*, *Rhus*, and soil of *Pinus* from Asia (India) and North America (Canada and USA: New York). See Kendrick et al. [4] for other details	Walker & Minter [5]
*G. bimorphosporum*	Brown, mononematous, unbranched, flexuous to erect, septate; terminal and intercalary nodose, distinctly echinulate conidiogenous regions; 175–550 × 6.2–10 µm;	Spherical to subspherical, polyblastic, terminal or intercalary, conidiogenous loci truncate, slightly raised; 13.5–45 × 15–22 µm	Pale to moderately brown, catenate, unicellular, of two types: primary conidia elongate-ellipsoid to elongate-obclavate, 7.5–11.5 × 3–4.5 µm; secondary conidia ellipsoid, smaller, 3–6.2 × 2.5–3.5 µm	Endophyte in leaves of *Carissa carandas*; India	Jacob & Bhat [14]
*G. fuscum*	Light to dark brown, slightly roughened, 250–2000 × 11–16 µm	Light to dark brown, slightly roughened, with swollen part, 28–38 × 23–38 µm	Light brown, catenate (chains of 2), ellipsoid, unicellular,, slightly roughened, 10–15 × 5–7 µm; denticles blackened	Rotting wood of *Quercus*; Italy. See Walker & Minter [5] for other reports	Walker & Minter [5]
*G. mangrovei*	Brown, macronematous, mononematous, solitary or grouped, unbranched, flexuous to erect, septate, paler toward the apex, nodose;(28–)115–215(–232) × (3–)4–7(–9) µm	Light to dark brown, polyblastic, indeterminate, integrated, terminal or intercalary, spherical to subspherical, with broad denticles; (4.5–)8–10(–11.5) × (4.5–)8–8.5(–10) µm	Hyaline when young to dark brown with age, catenate (branched and unbranched acropetal chains), globose to ellipsoid–cylindric; primary conidia, 4.5–5.5 × 3–4 µm; secondary conidia, (3–)4–4.5(–5.5) × (3–)3.5–4(–5) µm	Aureobasidium-like structures; Mangrove sediment; Brazil	This study
*G. parasiticum*	Hyaline to pale brown, smooth; 250–2000 × 9–15 µm	Hyaline to pale brown, smooth, with swollen region; 28–38 × 24–45 µm	Hyaline to pale brown, catenate (chains of 3), ellipsoid, unicellular, smooth or slightly roughened; 5–12 × 4–7 µm; denticles unblackened	On decaying fungi (*Hypocrea*, *Tremella*, and *Poria*); Europe and the USA. See Walker & Minter [5] for other reports	Walker & Minter [5]
*G. piceae*	Brown, short, simple; ca. 250 × 5–10 µm	Blastic, apical and/or lateral; cells not distinctly delimited; conidiogenous zones swollen, poroid, finely roughened; 9–16.5 µm diam (apical zones up to 18.5 µm)	Brown, unicellular, solitary, catenate (chains of 2–3); globose, 2.5–3 µm diam; ellipsoid, 6–9 × 2.5–3.5 µm; lacrymiform, reniform, fusiform, 9–16 × 3.5–5.5 µm; with a basal denticle	Fossil plantlet of *Picea* sp. in Baltic amber; Russia	Dörfelt & Schmidt [8]

**Fig. 4:**
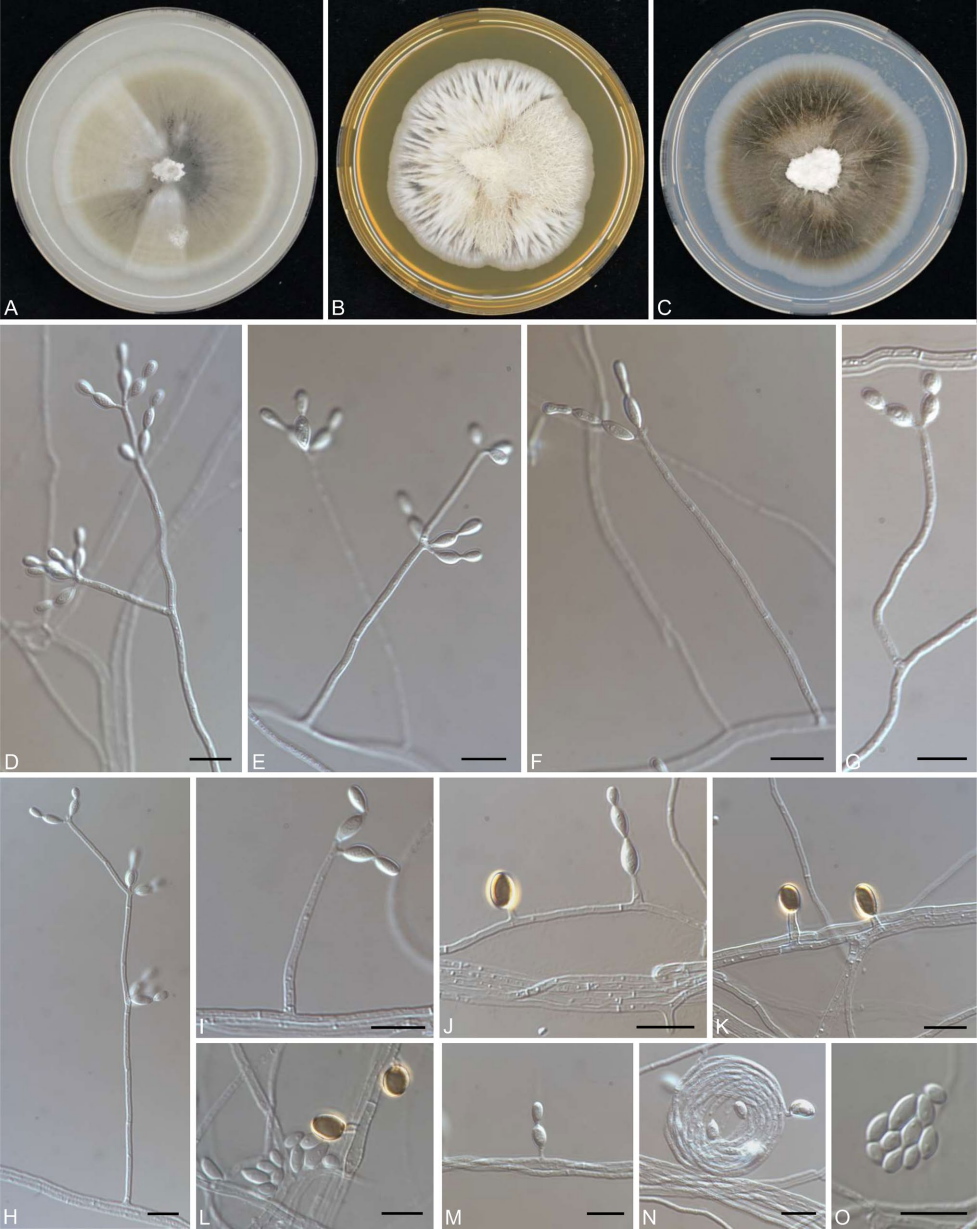
*Ophiostoma bahiense* comb. nov. (ex-type URM 339). **A**. Colony on OA. **B**. Colony on MEA. **C**. Colony on PDA. **D**-**I**. Conidiophores and conidia. **J**-**M**. Conidiogenous cells, conidia and aleuroconidia-like. **N**. Fertile coils and conidia. **O**. Conidia. Scale bars: 10 µm

***Ophiostoma bahiense*** (Bat.) J.D.P. Bezerra, ***comb. nov.*** Fig. 4.

*MycoBank:* MB826714.

*Basionym: Gonatobotryum bahiense* Bat., Anais Soc. Biol. Pernambuco 13(1): 154. 1955.

*Type:* **Brazil**, Bahia state, Salvador municipality, Instituto Biológico da Bahia, from the atmospheric air, November 7, 1955, *A. Domingues* (ex-type culture URM 339 = CBS 141532).

*Description:* On PDA. *Mycelium* septate, sometimes forming fertile coils, hyaline, 1.5 − 2.5 µm wide. *Conidiophores* erect or slightly curved, 0–2-septate, rarely 3-septate, unbranched or with conidiogenous cells sympodially branched, hyaline, (32 −) 65 − 124 (− 260) × 1.5 − 2.0 µm. *Conidiogenous cells* erect, arising directly from hyphae or reduced to hyphae, hyaline, (8–) 9.5 − 18.5 (–30.5) × 1.5 − 2.0 µm, and from conidiophores, proliferating sympodially, becoming denticulate, (1.5 −) 2.5 − 4.5 (− 7) × 1.5 − 2.0 µm. *Denticles* 0.5–1.5 μm long, usually in an apical crown of 2–3, scattered, solitary or in nodes. *Conidial secession* schizolytic. *Conidia* holoblastic, formed singly or in chains, non-septate, clavate, ovoid-obovoid, smooth, guttulate, thin-walled, hyaline, (3–) 5 − 6.5 (–7.5) × (2–) 2.5 − 3.5 (–5.5) µm, and aleuriconidia-like, non-septate, ovoid-obovoid, smooth, nonguttulate, brown, (6.5–) 7.5 − 8.5 (–9.5) × (4–) 5 − 5.5 (–7) µm. Sexual morph not observed.

*Culture characteristics:* Colonies on PDA are zonate, pale brown with white middle and cream rim, with sparse to moderate aerial mycelium, reverse colour similar to the surface, reaching 68 mm diam. On MEA, zonate, cream, merged with dark brown, with moderate aerial mycelium, reverse pale and merged with brown, growing up to 65 mm. On OA, zonate, light pale brown with grey middle and cream rim, slightly smooth surface, with moderate white aerial mycelium, growing up to 70 mm.

*Notes:* The strain URM 339 was circumscribed by Batista & Maia [15] as the type of *Gonatobotryum bahiense*. Later, Walker & Minter [5] excluded this species of the genus *Gonatobotryum* based on the dimensions of the narrow conidiophores. These authors did not check this ex-type strain and did not allocate it to another genus. In the present study, BLASTn searches of the ITS sequence revealed 92% and 91% identity to *Ophiostoma sparsiannulatum* (CMW17231; GenBank accession OM501515 and CBS 122815; GenBank accession MH863239, respectively), and 91% identity to *O. multiannulatum* (MUCL 19062; GenBank accession NR145274), among others. The LSU sequence showed 98% identity to *O. sparsiannulatum* (CMW17231; GenBank NG154007). The tubulin sequence has 91% identity to *O. palustre* (CMW 44403; GenBank KX273409). Phylogenetic analyses (Fig. 2) revealed that this strain is placed in the *O. multiannulatum* species complex, with *O. palustre* being a particularly close relative; therefore, we propose a new combination, *O. bahiense*, to accommodate the URM 339 strain. *Ophiostoma bahiense* was isolated from atmospheric air in Brazil, while *O. palustre* (ex-holotype CMW44403) was isolated from stem wounds of *Barringtonia racemosa* in South Africa [36]. Morphologically, *O. bahiense* can be distinguished from *O. palustre* by having hyphae forming fertile coils, longer conidiophores (up to 260 µm in *O. bahiense vs.* 110 µm in *O. palustre*), conidiogenous cells proliferating sympodially and becoming denticulate, in addition to producing holoblastic, aseptate, clavate to ovoid-obovoid conidia, and aleuriconidia-like.

### Excluded or doubtful species of ***Gonatobotryum*** (based on Walker & Minter [5])

***Gonatobotryum dichotomum*** Cooke & Massee, Grevillea 16(77): 15. 1887.

*Notes:* Walker & Minter [5] analysed the holotype and described it as “poor condition and the original description is inadequate”. Additionally, the authors treated *G. dichotomum* as a *nomen dubium*.

***Gonatobotryum indicum*** Munjal & H.S. Gill, Indian Phytopaphol. 16(1): 62. 1963.

*Notes:* Part of the holotype (HCIO 27078) was analysed by Walker & Minter [5], and no fungus was found similar to the original description and illustration. The authors treated it as a synonym of *G. fuscum* based on the description of Munjal & Gill [37].

***Gonatobotryum maculicola*** (G. Winter) Sacc., Syll. Fung. 4: 278. 1886.

*Notes:* In 1953, Hughes proposed the new combination *Gonatobotryum apiculatum* (Peck) Hughes and treated *G. maculicola* as its synonym. Later, Kendrick et al. [4] analysed 15 collections and identified them as *G. apiculatum*, following the classification proposed by Hughes [35]. On the other hand, Walker & Minter [5] treated *G. maculicola* as a synonym of *Gonatobotrys maculicola* G. Winter and explained that “There is no extant collection of this species in Winter’s herbarium in B”, suggested that the type is “apparently lost”, and treated *Gonatobotrys maculicola* as a *nomen dubium*.

***Gonatobotryum sclerotigenum*** Van Warmelo, Bothalia 10(2): 347. 1971.

*Notes:* Analysing the holotype (PRE 44252) of this species, Walker & Minter [5] highlighted that the “conidia appear to secede by schizolysis, and this combined with the presence of sclerotia”, suggesting a relation with *Botrytis* Mich. ex Pers. The authors did not place it definitely in this genus, but excluded the species from *Gonatobotryum*. Vu et al. [38], based on ITS-LSU rDNA sequence, treated the isotype strain CBS 261.71 (= IHEM 3835) of *G. sclerotigenum* as *B. cinerea* Pers.

***Gonatobotryum tenellum*** Peck, Bull. New York State Mus. 1(2): 17. 1887.

*Notes:* Although Walker & Minter [5] did not analyse the type material, the authors pointed out that “Peck’s description of the spores being produced in verticils of 2 − 4 at the septa of the conidiophores” was not congruent with *Gonatobotryum* or species of *Nematogonum* (*Incertae sedis*, *Helotiales*) and *Gonatobotrys* (*Ceratostomataceae*, *Melanosporales*) [3]. Walker & Minter [5] excluded it from *Gonatobotryum*, treating it as *incertae sedis*.

## Discussion

In this study, we analysed fresh *Gonatobotryum* isolates from mangrove sediment and assessed the type strain of *G. bahiense* (URM 339), studied by Batista & Maia [15] in Brazil. Based on morphological observations and multi-locus phylogenetic analyses, we introduced a new species, *G. mangrovei*. *Gonatobotryum bahiense* (URM 339) was transferred to *Ophiostoma*, and *O. bahiense* was proposed as a new combination. The genus *Gonatobotryum* is reported for the first time from a mangrove sediment in Brazil.

*Gonatobotryum* is a genus of anamorphic fungi characterised by species that occur predominantly associated with other fungi or plant substrates [5]. The species have been reported as mycoparasites, saprophytes, and on plant material, occurring in a wide variety of habitats, including forest soils, decaying woody substrates, filamentous fungi, living plant tissues, and fossil plant material preserved in amber [13, 14]. These records indicate considerable ecological versatility within the genus.

Species of *Gonatobotryum* have traditionally been classified exclusively on morphological characters [5]. Molecular data are unavailable from the type material of the genus. Even so, species in this genus remain morphologically recognisable on the basis of a consistent combination of characters [5]. This study demonstrates that integrating morphological and molecular data enables the delimitation and classification of species in *Gonatobotryum*, clarifying the taxonomy of old genera known solely from morphological features, as previously adopted for *Periconia* [39] and supported by broader conceptual discussions in fungal systematics [40]. The reference DNA sequences analysed in this study should prove valuable for future studies on *Gonatobotryum*.

We observed that *G. mangrovei* exhibits an aureobasidium-like morphology, similar to that described by Gao et al. [9] in an isolate identified as “*G. apiculatum*” and here treated as *Gonatobotryum* sp. In most descriptions of *Gonatobotryum* species, no distinction was made among ramoconidia, primary, or secondary conidia. We decided to use these terminologies because we can clearly see these conidial differences in our fresh material and in the previously described species in this genus. Similarly, Cole [12] studied the ultrastructure of conidiogenesis in *G. apiculatum* and described primary conidia arising from conidiogenous cells and secondary conidia arising from preexisting conidia; this terminology is similar to that used here. Walker & Minter [5] observed in *G. apiculatum* the production of “distal conidia being smaller than proximal ones”, suggesting the same as we are describing here. Another notable morphological feature was the production of aureobasidium-like structures in our isolates. Gao et al. [9] explained that “numerous conidia were gathered on the base of the colony on PDA showing pus-like heap” when studying an isolate of *Gonatobotryum* sp. (YZU 181227) causing leaf spot and blight disease in China. Gao et al. [9] also highlighted that all “those conidia were directly produced from hyphae on the surface of the medium”, being the first observation of this type of conidia in *Gonatobotryum*.

Batista & Maia [15] originally circumscribed URM 339 as the ex-type of *Gonatobotryum bahiense*. Walker & Minter [5] later excluded the species from *Gonatobotryum* based on morphology but left it without a generic placement. Our results resolve this long-standing uncertainty, demonstrating that the ex-type strain is phylogenetically and morphologically aligned with *Ophiostoma*. This genus contains important plant pathogens affecting trees and agronomic crops and is disseminated by some *Coleoptera* [41–45]. Species in this genus also have medical importance and mainly occur in the Northern Hemisphere [46, 47]. Furthermore, several authors have studied the taxonomy of *Ophiostoma* to clarify its relationships with closely related genera [21, 36, 48–50].

This study introduced a new anamorphic species from mangrove sediment, reinforcing the idea that mangrove ecosystems are important reservoirs of fungal diversity [51, 52], with specific potential to reveal new taxa and provide new perspectives on morphological and molecular diversity of *Ascomycota*. This study expands the known geographic distribution of *Gonatobotryum* species associated with tropical mangroves and contributes to a clearer understanding of their taxonomy, ecology, and evolutionary relationships, highlighting the importance of combining morphological and molecular analysis.

## Supplementary Information

Below is the link to the electronic supplementary material.

Supplementary file1 Online Resource 1 Maximum Likelihood (ML) phylogenetic tree based on ITS sequences. The new species, *Gonatobotryum mangrovei* sp. nov., is shown in bold among related taxa within the family *Dothideaceae (Dothideales)*. Support values for ML-BS and IQ-TREE-BS ≥ 70% (UFboot2/RAxML) and BPP ≥ 0.95 are indicated near the nodes, and (–) indicates support below the threshold values. Ex-type strains are marked with (T). The tree was rooted to *Phaeosclera dematioides* (CBS 157.81) (*Phaeosclerales, Dothideomycetes*). (PDF 548 KB)

Supplementary file2 Online Resource 2 Maximum Likelihood (ML) phylogenetic tree based on LSU sequences. The new species, *Gonatobotryum mangrovei* sp. nov., is shown in bold among related taxa within the family *Dothideaceae (Dothideales)*. Support values for ML-BS and IQ-TREE-BS ≥ 70% (UFboot2/RAxML) and BPP ≥ 0.95 are indicated near the nodes, and (–) indicates support below the threshold values. Ex-type strains are marked with (T). The tree was rooted to *Phaeosclera dematioides* (CBS 157.81) (*Phaeosclerales, Dothideomycetes*). (PDF 528 KB)

## Data Availability

The data supporting the findings of this study are available on Figshare, in GenBank, and in mycological collections (details above).
