# Supplementary material for: Morphological and phylogenetic analyses of Gonatobotryum (Dothideaceae, Dothideales), and the description of a new species from a mangrove sediment in Brazil
Source: Braz J Microbiol. 2026 Aug 18;57(1):243. doi: 10.1007/s42770-026-02052-1 (PMC13486461; doi:10.1007/s42770-026-02052-1)
Supplement: Supplementary file 1 [file 42770_2026_2052_MOESM1_ESM.pdf]

Morphological and phylogenetic analyses of *Gonatobotryum* (*Dothideaceae*, Dothideales), and the description of a new species from a mangrove sediment in Brazil

Brazilian Journal of Microbiology

Karen B.E. Carmo[1], Thiago G. Carvalho[1,2], Camila S. Oliveira[1,2], Esmael Alves Vital[5], Gabriel G. Barreto[6], Luis F.P. Gusmão[7], Melissa F. Landell [5], Pedro H. Félix-Oliveira [1,2], Cristina M. Souza-Motta [3,4], Layanne O. Ferro [1,2], Jadson D.P. Bezerra[1,2,4]

[1] Laboratório de Micologia (LabMicol), Instituto de Patologia Tropical e Saúde Pública, Universidade Federal de Goiás, Rua 235, s/n, Setor Universitário, 74605-050, Goiânia, GO, Brazil.

[2] Programa de Pós-Graduação em Biologia da Relação Parasito-Hospedeiro (PPGBRPH), Instituto de Patologia Tropical e Saúde Pública, Universidade Federal de Goiás, Rua 235, s/n, Setor Universitário, 74605-050, Goiânia, GO, Brazil.

[3] Departamento de Micologia Prof. Chaves Batista, Universidade Federal de Pernambuco, Av. Prof. Moraes Rego, s/n, Centro de Biociências, Cidade Universitária, 50670-901, Recife, PE, Brazil.

[4] Programa de Pós-Graduação em Biologia de Fungos (PPGBF), Departamento de Micologia Prof. Chaves Batista, Universidade Federal de Pernambuco, Av. Prof. Moraes Rego, s/n, Centro de Biociências, Cidade Universitária, 50670-901, Recife, PE, Brazil.

[5] Instituto de Ciências Biológicas e da Saúde, Universidade Federal de Alagoas, Campus A.C. Simões, 57072-900, Maceió, AL, Brazil.

[6] Universidade Federal da Paraíba, Centro de Ciências Agrárias - Campus II, Departamento de Biociências, Rodovia PB 079 - Km 12, 58397-000, Areia, Paraíba, Brazil.

[7] Universidade Estadual de Feira de Santana, Departamento de Ciências Biológicas, Av. Transnordestina s/n, Novo Horizonte, 44036-900, Feira de Santana, Bahia, Brazil.

Correspondence: L.O. Ferro (layanne.ferro93@gmail.com) and J.D.P. Bezerra (jadsonbezerra@ufg.br).

Online Resource 1 Maximum Likelihood (ML) phylogenetic tree based on ITS sequences. The new species, *Gonatobotryum mangrovei* sp. nov., is shown in bold among related taxa within the family *Dothideaceae* (Dothideales). Support values for ML-BS and IQ-TREE-BS ≥ 70% (UFboot2/RAxML) and BPP ≥ 0.95 are indicated near the nodes, and (–) indicates support below the threshold values. Ex-type strains are marked with (T). The tree was rooted to *Phaeosclera dematioides* (CBS 157.81) (Phaeosclerales, Dothideomycetes).

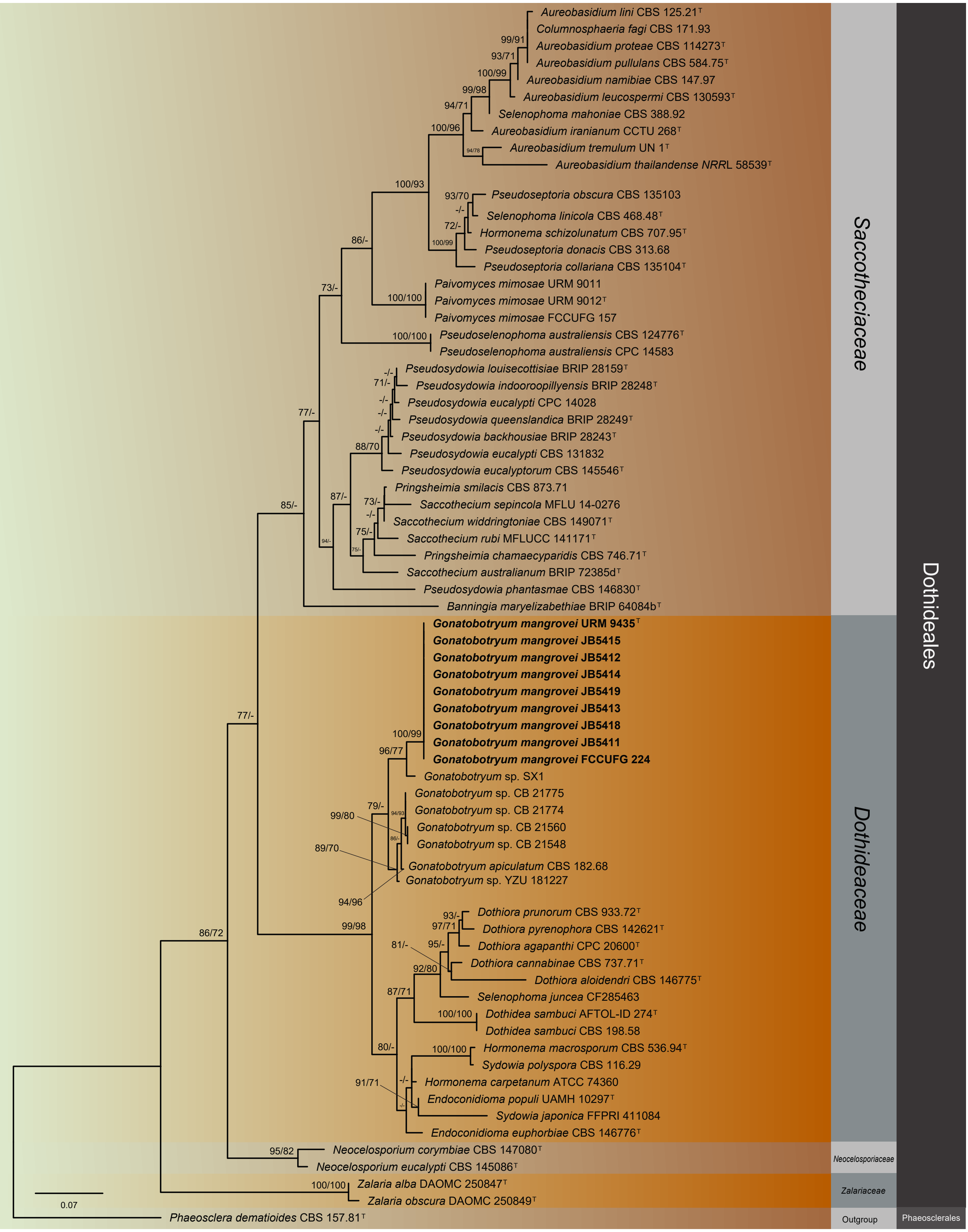

Aureobasidium lini CBS 125.21T
Columnosphaeria fagi CBS 171.93
Aureobasidium proteae CBS 114273T
Aureobasidium pullulans CBS 584.75T
Aureobasidium namibiae CBS 147.97
Aureobasidium leucospermi CBS 130593T
Selenophoma mahoniae CBS 388.92
Aureobasidium iranianum CCTU 268T
Aureobasidium tremulum UN 1T
Aureobasidium thailandense NRRL 58539T
Pseudoseptoria obscura CBS 135103
Selenophoma linicola CBS 468.48T
Hormonema schizolunatum CBS 707.95T
Pseudoseptoria donacis CBS 313.68
Pseudoseptoria collariana CBS 135104T
Paivomyces mimosae URM 9011
Paivomyces mimosae URM 9012T
Paivomyces mimosae FCCUFG 157
Pseudoselenophoma australiensis CBS 124776T
Pseudoselenophoma australiensis CPC 14583
Pseudosydowia louisecottisiae BRIP 28159T
Pseudosydowia indooroopillyensis BRIP 28248T
Pseudosydowia eucalypti CPC 14028
Pseudosydowia queenslandica BRIP 28249T
Pseudosydowia backhousiae BRIP 28243T
Pseudosydowia eucalypti CBS 131832
Pseudosydowia eucalyptorum CBS 145546T
Pringsheimia smilacis CBS 873.71
Saccothecium sepincola MFLU 14-0276
Saccothecium widdringtoniae CBS 149071T
Saccothecium rubi MFLUCC 141171T
Pringsheimia chamaecyparidis CBS 746.71T
Saccothecium australianum BRIP 72385dT
Pseudosydowia phantasmae CBS 146830T
Banningia maryelizabethiae BRIP 64084bT
Gonatobotryum mangrovei URM 9435T
Gonatobotryum mangrovei JB5415
Gonatobotryum mangrovei JB5412
Gonatobotryum mangrovei JB5414
Gonatobotryum mangrovei JB5419
Gonatobotryum mangrovei JB5413
Gonatobotryum mangrovei JB5418
Gonatobotryum mangrovei JB5411
Gonatobotryum mangrovei FCCUFG 224
Gonatobotryum sp. SX1
Gonatobotryum sp. CB 21775
Gonatobotryum sp. CB 21774
Gonatobotryum sp. CB 21560
Gonatobotryum sp. CB 21548
Gonatobotryum apiculatum CBS 182.68
Gonatobotryum sp. YZU 181227
Dothiora prunorum CBS 933.72T
Dothiora pyrenophora CBS 142621T
Dothiora agapanthi CPC 20600T
Dothiora cannabinae CBS 737.71T
Dothiora aloidendri CBS 146775T
Selenophoma juncea CF285463
Dothidea sambuci AFTOL-ID 274T
Dothidea sambuci CBS 198.58
Hormonema macrosporum CBS 536.94T
Sydowia polyspora CBS 116.29
Hormonema carpetanum ATCC 74360
Endoconidioma populi UAMH 10297T
Sydowia japonica FFPRI 411084
Endoconidioma euphorbiae CBS 146776T
Neocelosporium corymbiae CBS 147080T
Neocelosporium eucalypti CBS 145086T
Zalaria alba DAOMC 250847T
Zalaria obscura DAOMC 250849T
Phaeosclera dematioides CBS 157.81T
0.07
Saccotheciaceae
Dothideaceae
Neocelosporiaceae
Zalariaceae
Outgroup
Dothideales
Phaeosclerales
